# Blockade of LFA-1 augments in vitro differentiation of antigen-induced Foxp3^+^ Treg cells

**DOI:** 10.1016/j.jim.2014.07.012

**Published:** 2014-12-01

**Authors:** Johan Verhagen, David C. Wraith

**Affiliations:** School of Cellular and Molecular Medicine, University of Bristol, Bristol, United Kingdom

**Keywords:** Foxp3, LFA-1, Treg cell, Immunotherapy, Autoimmunity

## Abstract

Adoptive transfer of antigen-specific, in vitro-induced Foxp3^+^ Treg (iTreg) cells protects against autoimmune disease. To generate antigen-specific iTreg cells at high purity, however, remains a challenge. Whereas polyclonal T cell stimulation with anti-CD3 and anti-CD28 antibody yields Foxp3^+^ iTreg cells at a purity of 90–95%, antigen-induced iTreg cells typically do not exceed a purity of 65–75%, even in a TCR-transgenic model. In a similar vein to thymic Treg cell selection, iTreg cell differentiation is influenced not only by antigen recognition and the availability of TGF-β but also by co-factors including costimulation and adhesion molecules. In this study, we demonstrate that blockade of the T cell integrin Leukocyte Function-associated Antigen-1 (LFA-1) during antigen-mediated iTreg cell differentiation augments Foxp3 induction, leading to approximately 90% purity of Foxp3^+^ iTreg cells. This increased efficacy not only boosts the yield of Foxp3^+^ iTreg cells, it also reduces contamination with activated effector T cells, thus improving the safety of adoptive transfer immunotherapy.

## Highlights

• iTreg cells can be generated in an antigen-specific manner, even if specific Tconv cells are present at low frequency.
• Blockade of anti-LFA-1 during iTreg cell differentiation augments Foxp3 induction.
• The blockade of LFA-1 alters the iTreg cell phenotype but does not impair stability or function.

## 1 Introduction

In addition to thymic regulation, peripheral induction of a regulatory phenotype in conventional T (Tconv) cells provides protection from undesirable immune responses to self antigens. Adoptive transfer of in-vitro induced Foxp3^+^ T regulatory (iTreg) cells forms an attractive approach to therapeutically restore the balance when healthy immunity is disturbed, i.e. in autoimmune disease. iTreg cells can be generated at very high purity by polyclonal stimulation of naive CD4^+^CD62L^+^ T cells with anti-CD3 and anti-CD28 antibody in the presence of TGF-β and IL-2 (Thornton et al., 2010, Verhagen et al., 2013a). Studies using iTreg cells obtained in this way from TCR-transgenic mice have demonstrated that antigen-specificity is an important factor in the functionality of transferred iTreg cells (DiPaolo et al., 2007, Chattopadhyay and Shevach, 2013) (personal observation JV, unpublished). Although iTreg cells have been the subject of intense investigation, the in vitro differentiation of antigen-specific iTreg cells, using cognate ligand rather than anti-CD3 and anti-CD28, at high purity remains a challenge. We have demonstrated previously that, in the Tg4 mouse model, iTreg cells can be generated using cognate Myelin Basic Protein (MBP) peptide as a stimulus but the level of conversion lags behind that achieved using antibody stimulation (65–75% vs 90–95%) (Verhagen et al., 2013a). This low purity not only limits the yield of Foxp3^+^ iTreg cells, the contamination with activated Foxp3^−^ T cells that may exert pro-inflammatory effector functions poses a potential risk when used for Treg cell-based immunotherapy.

Development of Foxp3^+^ iTreg cells depends not only on TCR signals and the availability of TGF-β, but also on additional co-factors. For example, CTLA-4 has previously been suggested to be important for TGF-β-dependent Foxp3 induction (Zheng et al., 2006), although this finding was recently contradicted (Chattopadhyay and Shevach, 2013). In this study, we compared the effects of ligation or blockade of a number of costimulatory and adhesion molecules involved in T cell activation and regulation on Foxp3 induction in vitro. Interestingly, we found that blockade of Leukocyte Function-associated Antigen-1 (LFA-1) with monoclonal antibody augmented antigen-induced Foxp3 expression, giving rise to iTreg cells approximately 90% Foxp3^+^. LFA-1 is an integrin that, through interaction with its ligand ICAM-1, mediates T cell-APC interaction and is involved in stable formation of the immunological synapse (Shimizu, 2003). It, therefore, controls the avidity of the activation signals received through the T cell receptor and costimulatory molecules. Although signaling through LFA-1 itself is less well defined, LFA-1 ligation has been shown to make CD4^+^ T cells refractory to TGF-β signaling through upregulation of Smad7, SKI and SMURF2 (Verma et al., 2012). We have not been able to establish if the effect of anti-LFA-1 during iTreg differentiation follows a direct or indirect impact of LFA-1 on Foxp3 induction but the result is in line with previous findings; the prevention of allogeneic transplant rejection by treatment with anti-LFA-1 has been shown to be associated with an increased frequency of CD4^+^Foxp3^+^ Treg cells in the graft-draining lymph nodes (Reisman et al., 2011). Here, we demonstrate that our method induces antigen-specific iTreg cells of high purity that successfully protect against CNS autoimmune disease.

## 2 Materials and methods

### 2.1 Mice

B10.PL, Tg4, Tg4 CD45.1^+^ and Tg4 Foxp3^gfp^ (Verhagen et al., 2013a) mice were bred and kept under specific pathogen-free conditions. All experiments were carried out under a UK Home Office Project Licence and were subject to assessment by the University of Bristol ethical review committee.

### 2.2 Peptide

The acetylated N-terminal peptide of murine MBP, Ac1-9 (Ac-ASQKRPSQR) and its high MHC affinity variant (Ac-ASQYRPSQR) were custom synthesized (purity > 85%; GL Biochem (Shanghai) Ltd.)

### 2.3 Naive T cell isolation

CD4^+^CD62L^+^ naive T cells were isolated magnetically from splenocytes using a naive T cell isolation kit (Stemcell Technologies) according to the manufacturer's recommendations.

### 2.4 iTreg cell differentiation

CD4^+^CD62L^+^ naive splenic T cells were cultured in vitro for 7 days in RPMI medium supplemented with 5% FCS, in the presence of 100 U/ml rhIL-2 (R&D systems) and 10 ng/ml rhTGF-β_1_ (Peprotech). Cells were stimulated either with anti-CD3e (1 μg/ml) and anti-CD28 (2 μg/ml) plate-bound antibody (both from eBioscience) or MBP Ac1-9 peptide in the presence of irradiated B10.PL splenocytes used as antigen-presenting cells. Where indicated, functional grade antibody to LFA-1 (M17/4, Biolegend or eBioscience), CTLA-4 (9H10, eBioscience), PD-1 (J43, BioXCell), LAG3 (C9B7W, BioXCell) or IL-10R (1B1.3A, BioXCell) was added either plate-bound or soluble in the medium at 10 μg/ml for the duration of the culture. The level of FoxP3 induction was assessed by flow cytometry.

### 2.5 Flow cytometry

Flow cytometric analysis was performed using an LSR II or Fortessa X20 flow cytometer (BD). Cell phenotypes were analyzed using combinations of anti-FoxP3-PE, − efluor450 or –APC, anti-CD45.2-PerCPCy5.5, anti-CD45.1 PE-Cy7, anti-CD62L-PE-Cy7, anti-Ki67-ef450, anti-CD4-AlexaFluor700 (all from eBioscience), anti-Neuropilin-1-PE or − APC, anti-LFA-1 (clone 2D7)-PE, anti-Helios-FITC, and anti-CD103-PerCPCy5.5 (all from Biolegend) antibodies. Fixable viability dye eFluor780 (eBioscience) was used in all experiments to exclude dead cells. Cell proliferation dye-ef450 (CPD-ef450, eBioscience) was used to visualize cell divisions or calculate division and proliferation indexes. Results were analyzed using FlowJo analysis software (Tree Star, Inc.).

### 2.6 Demethylation analysis

Demethylation analysis of the foxp3 CNS2 region was carried out by EpigenDX, assay ADS568. The heat map matrix was created using the free online tool on http://www.chibi.ubc.ca/matrix2png/bin/matrix2png.cgi. Each block in the heat map represents the mean of 3 individual samples per condition. Female mice were used for the analysis. Therefore, the level of methylation is relative, with the highest level of methylation in any CpG in the CD4^+^ Tconv cell group set as the maximum and the lowest level in any CpG in the CD4^+^CD25^+^ Treg cell group as the minimum.

### 2.7 Induction and scoring of EAE

Tg4 mice, with 5 × 10^6^ iTreg cells in PBS or PBS only transferred i.p. on day − 3, were primed subcutaneously at the base of the tail with 200 μg of MBP Ac1-9 in 0.1 ml of a sonicated emulsion consisting of an equal volume of complete Freund's adjuvant (CFA) and PBS, containing 4 mg/ml of heat-killed *Mycobacterium Tuberculosis* (both from Difco). On days 0 and 2, 200 ng of Pertussis toxin (Sigma Aldrich) was administered intraperitoneally in 0.5 ml of PBS. EAE was assessed twice daily with the following scoring system: 0, no signs; 1, flaccid tail; 2; impaired righting reflex and/or gait; 3, hind limb paralysis; 4, forelimb and hind limb paralysis; 5, moribund.

### 2.8 Statistical analysis

Data were analyzed for statistical significance using GraphPad Prism software.

## 3 Results and discussion

### 3.1 Foxp3 expression can be induced with peptide even in low-frequency antigen-specific Tconv cells

**Fig. 1:**
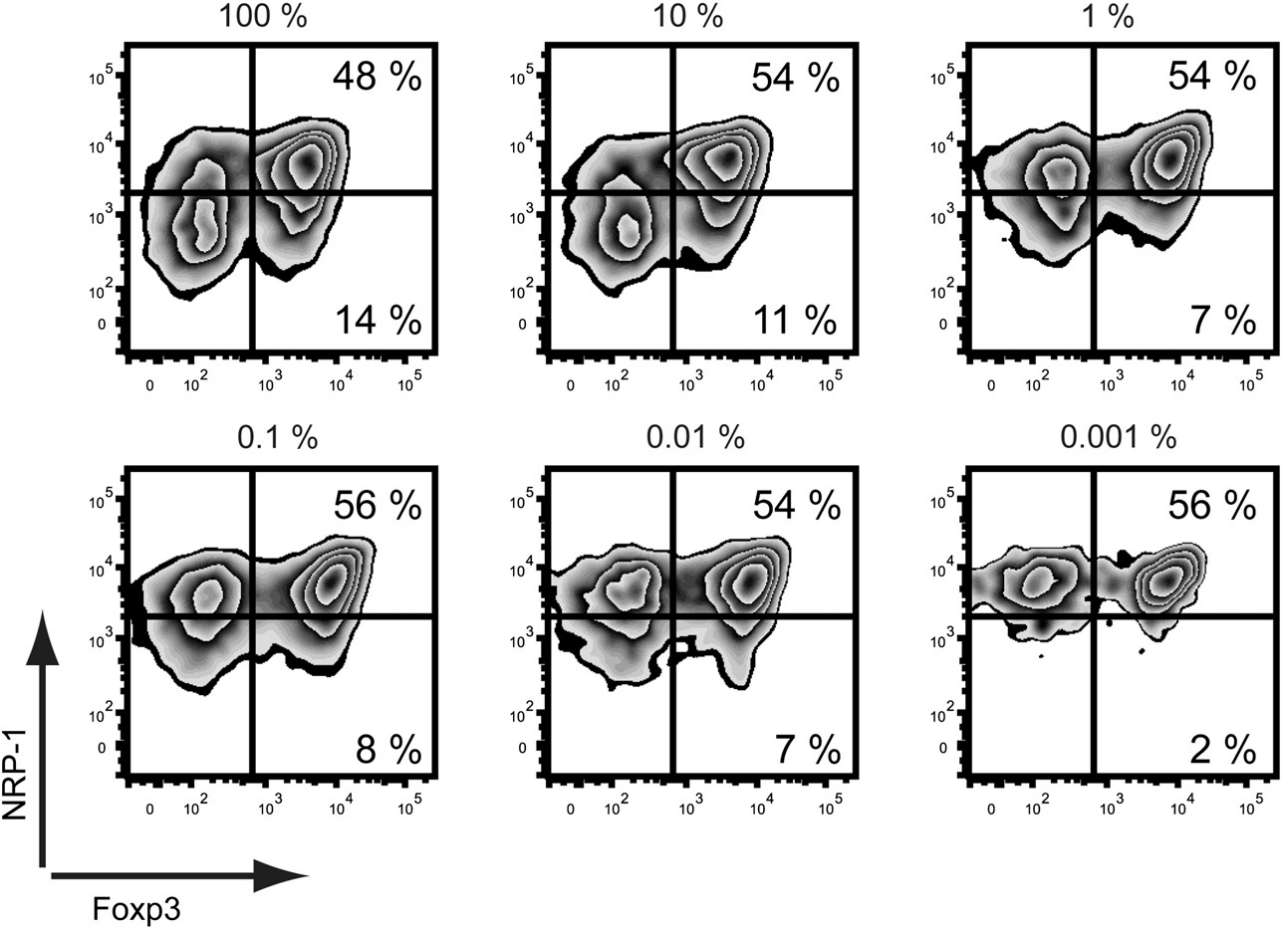
Foxp3 expression can be induced in antigen-specific Tconv cells with peptide, even when at low frequency. Tg4 CD45.1^+^ naive CD4^+^ T cells were titrated down among B10.PL (CD45.2^+^) naive T cells (from 100 down to 0.001% TCR-transgenic) before Foxp3 induction by activation with MBP Ac1-9 (1 μg/ml) in the presence of IL-2 and TGF-β_1_. Plots gated on live CD45.1^+^ cells on day 7 of differentiation culture. 1 experiment representative of 3 is shown.

In experimental settings, antigen-specific iTreg cells are commonly generated from murine TCR-transgenic CD4^+^ T cells through activation with plate-bound anti-CD3 and anti-CD28 antibodies in the presence of TGF-β and IL-2 since this method generates large numbers of Foxp3^+^ cells at very high purity (Thornton et al., 2010, Verhagen et al., 2013a). Although this method is well suited to investigating the function of antigen-specific iTreg cells in various settings, it obviously cannot be used to generate antigen-specific iTreg cells in a polyclonal system. We previously showed in the Tg4 mouse model, where > 90% of CD4^+^ T cells recognize the MBP Ac1-9 peptide, that Foxp3 can be induced in Tconv cells by stimulation with cognate peptide in the presence of irradiated APCs, TGF-β and IL-2 (Verhagen et al., 2013a). To demonstrate that antigen-specific Tconv cells in a polyclonal system, where their frequency will be much lower, can still successfully be differentiated into iTreg cells, CD4^+^CD62L^+^CD45.1^+^ Tg4 T cells were titrated among non-transgenic naive B10.PL CD45.2^+^ T cells down to 1 TCR-transgenic T cell in 100,000 and stimulated with 1 μg/ml MBP Ac1-9 in the presence of IL-2 and TGF-β. Even at the lowest ratio, antigen-specific Tg4 CD45.1^+^ T cells upregulated Foxp3 expression as effectively as when all T cells were TCR transgenic, although the frequency of Foxp3^+^ cells remained relatively low (Fig. 1). Clearly, the number of single antigen-specific iTreg cells retrieved at the end of the differentiation culture will be limited in a polyclonal system. Optimization of the rate of Foxp3 induction in antigen-specific T cells was therefore required.

### 3.2 Anti-LFA-1 augments Foxp3 induction during iTreg cell differentiation

**Fig. 2:**
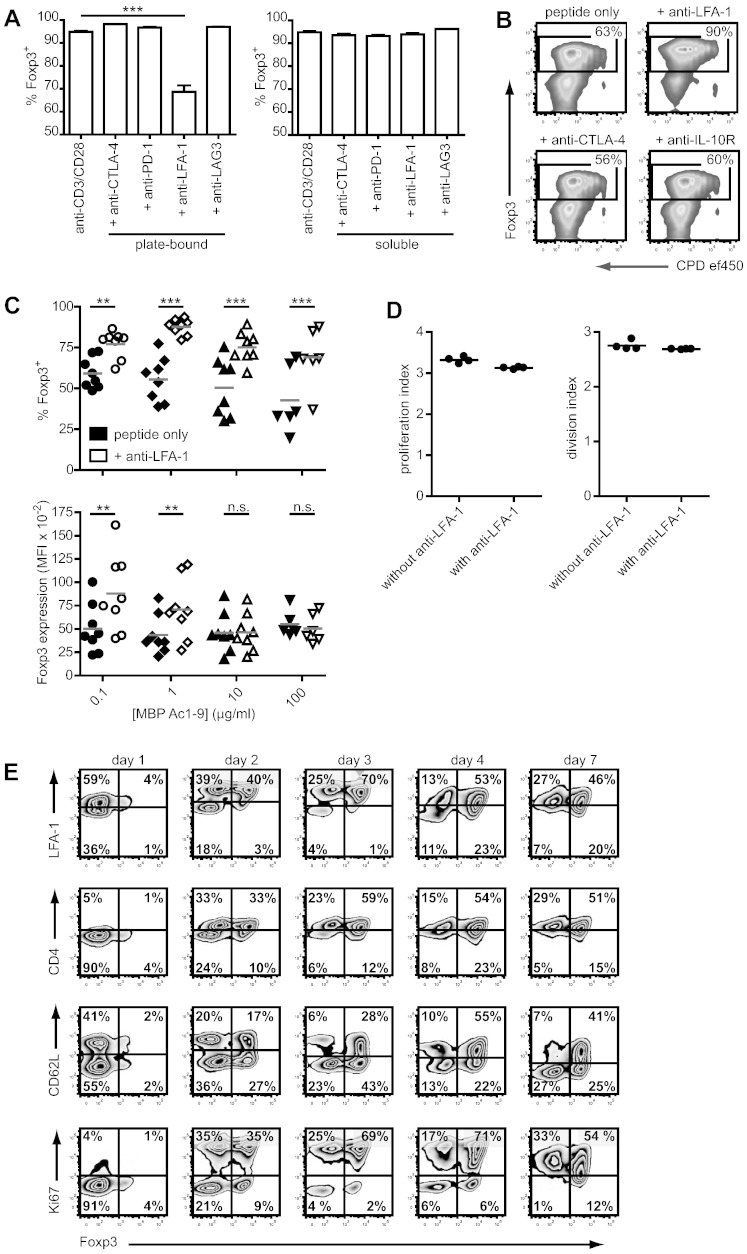
LFA-1 modulates antigen-specific Foxp3 induction. A: Foxp3 induced in CD4^+^CD62L^+^ Tg4 T cells on plates coated with anti-CD3 and anti-CD28, with or without anti-CTLA-4, − PD-1, − LFA-1 or -LAG3, either plate-bound or soluble (all at 10 μg/ml). Mean of triplicates + SEM. Each bar is representative of 3 or more similar experiments. ***P < 0.001, One-way ANOVA with Dunnett's post-test. B: Foxp3 induction in and proliferation of CPD-ef450-labeled CD4^+^ Tg4 T cells stimulated with 1 μg/ml MBP Ac1-9 and irradiated APC, in the presence or absence of soluble anti-LFA-1, − CTLA-4 or -IL-10R at 10 μg/ml. Representative of three similar experiments. C: Foxp3 induction in CD4^+^CD62L^+^ Tg4 T cells after 7-day culture with titrated amounts of MBP Ac1-9 and irradiated APC, with or without 10 μg/ml soluble anti-LFA-1. Top; percentage of Foxp3^+^ cells among live CD4^+^ cells. Bottom; median fluorescence of Foxp3 staining, gated on CD4^+^Foxp3^+^ cells. n = 6–8 individual experiments per condition. **P < 0.01, ***P < 0.001,2-tailed paired t-test. D: Proliferation and division indexes of T cells labeled with CPD-ef450 after 7-day iTreg cell differentiation culture, stimulated with 1 μg/ml MBP Ac1-9 with or without 10 μg/ml anti-LFA-1 in the culture. Gated on live CD4^+^ Foxp3^+^ cells. Differences not significant, unpaired, 2-tailed t-test. E: LFA-1^hi^, CD4^hi^, CD62L, Ki67 and Foxp3 expression on live CD4^+^ T cells during 7-day iTreg cell differentiation culture, stimulated with 1 μg/ml MBP Ac1-9 and irradiated APC.

The induction of Foxp3 expression during thymic selection is governed not only by the strength of TCR ligation, but also by cytokines and co-factors including adhesion molecules and co-stimulation (Verhagen et al., 2013b). In an attempt to enhance Foxp3 induction in vitro, the effect of several co-factors on iTreg cell differentiation was therefore examined. First, the effect of antibodies to CTLA-4 (clone 9H10), PD-1 (clone J43), LFA-1 (CD11a, clone M17/4) and LAG3 (clone C9B7W), all at 10 μg/ml and either plate-bound or soluble, on Foxp3 induction in CD4^+^ T cells stimulated with anti-CD3 and anti-CD28 was assessed. As depicted in Fig. 2A, ligation of LFA-1 with plate-bound antibody significantly reduced Foxp3 expression, whereas none of the other antibodies had a significant effect on Foxp3 induction. In the next step, the effect of soluble antibody to LFA-1, CTLA-4 or IL-10R (clone 1B1.3A) on antigen-induced Foxp3 expression was assessed. As expected from the opposite effect of plate-bound anti-LFA-1 on antibody-mediated iTreg cell differentiation, this demonstrated that blockade of LFA-1 with soluble antibody dramatically augmented Foxp3 induction in Tg4 Tconv cells (Fig. 2B). In contrast, blockade of CTLA-4 had only a modest inhibitory effect, while no consistent effect of IL-10R blockade was observed. Although LFA-1 activation is linked to CTLA-4 signaling (Schneider et al., 2005), in our system the reduction in Foxp3 expression in CTLA-4 deficient iTreg cells could not be reversed using anti-LFA-1 (not shown). This is in line, however, with the synergistic effects of anti-LFA-1 and CTLA-4Ig observed in the inhibition of transplant graft rejection (Reisman et al., 2011).

Considering the role of LFA-1 in the stable formation of the immunological synapse and therefore the avidity of the T cell-APC interaction, the effect of blockade of LFA-1 was hypothesized to be tied to the strength of antigenic stimulation. To assess this, CD4^+^CD62L^+^ Tg4 T cells were stimulated with a titrated dose range of MBP Ac1-9 with or without soluble anti-LFA-1 in the medium before determining the percentage of Foxp3 expressing cells as well as the mean level of Foxp3 expression per cell (as expressed by the median fluorescence index (MFI)) on day 7. Anti-LFA-1 significantly augmented the percentage of Foxp3-expressing cells over the range of peptide concentrations tested but peaked at 1 μg/ml of peptide, where the mean frequency of Foxp3 expression reached 87.6 ± 1.7%, n = 8 (Fig. 2C). The level of Foxp3 expression per cell was increased significantly only at lower peptide concentrations, which could be important as Foxp3 stabilizes its own expression (Gavin et al., 2007). Without the addition of anti-LFA-1, the concentration of peptide in the culture correlated inversely to the level of Foxp3 expression. Further lowering of the peptide concentration below 0.1 μg/ml, however, did not sufficiently stimulate the naive T cells in culture and, thus, did not give rise to sizeable numbers of viable iTreg cells (not shown). Despite the interference of stable immunological synapse formation that will result from the inhibition of LFA-1 binding to ICAM-1, the proliferation of CD4^+^ T cells over 7 days under iTreg cell differentiation conditions is not impaired (Fig. 2D), resulting in a high yield of iTreg cells. The blockade of LFA-1, therefore, does not exert its effect by merely lowering the TCR signal but actively changes the signaling involved in Foxp3 induction. This may involve the blockade of LFA-1-mediated upregulation of Smad7, SKI and SMURF2 that renders CD4^+^ T cells refractory to TGF-β (Verma et al., 2012).

To gain a greater insight into the role of LFA-1 during iTreg cell differentiation, its expression was assessed daily during the 7-day culture. As shown in Fig. 2E, although LFA-1 was expressed on all CD4^+^ T cells, the level of expression was differentially regulated on Foxp3^−^ and Foxp3^+^ cells at the early stages of antigen-mediated iTreg cell differentiation, correlating with changes in the expression levels of CD4, CD62L and the marker of cell division, Ki67. This could relate to the activation status of the cells but, tantalizingly, this unequal distribution of LFA-1, in conjunction with the TCR co-receptor CD4 and coinciding with differential T cell proliferation, is also reminiscent of the recently described phenomenon of asymmetric cell division (Chang et al., 2007, King et al., 2012). However, a role for this process in iTreg cell differentiation is not supported by the limited effect of variations in antigenic strength observed in conditions with anti-LFA-1 (Fig. 2C). A direct effect of LFA-1 blockade on susceptibility to TGF-β signaling (Verma et al., 2012) may, therefore, be the more likely explanation.

### 3.3 Anti-LFA-1 treatment alters the phenotype but not stability or function of iTreg cells

**Fig. 3:**
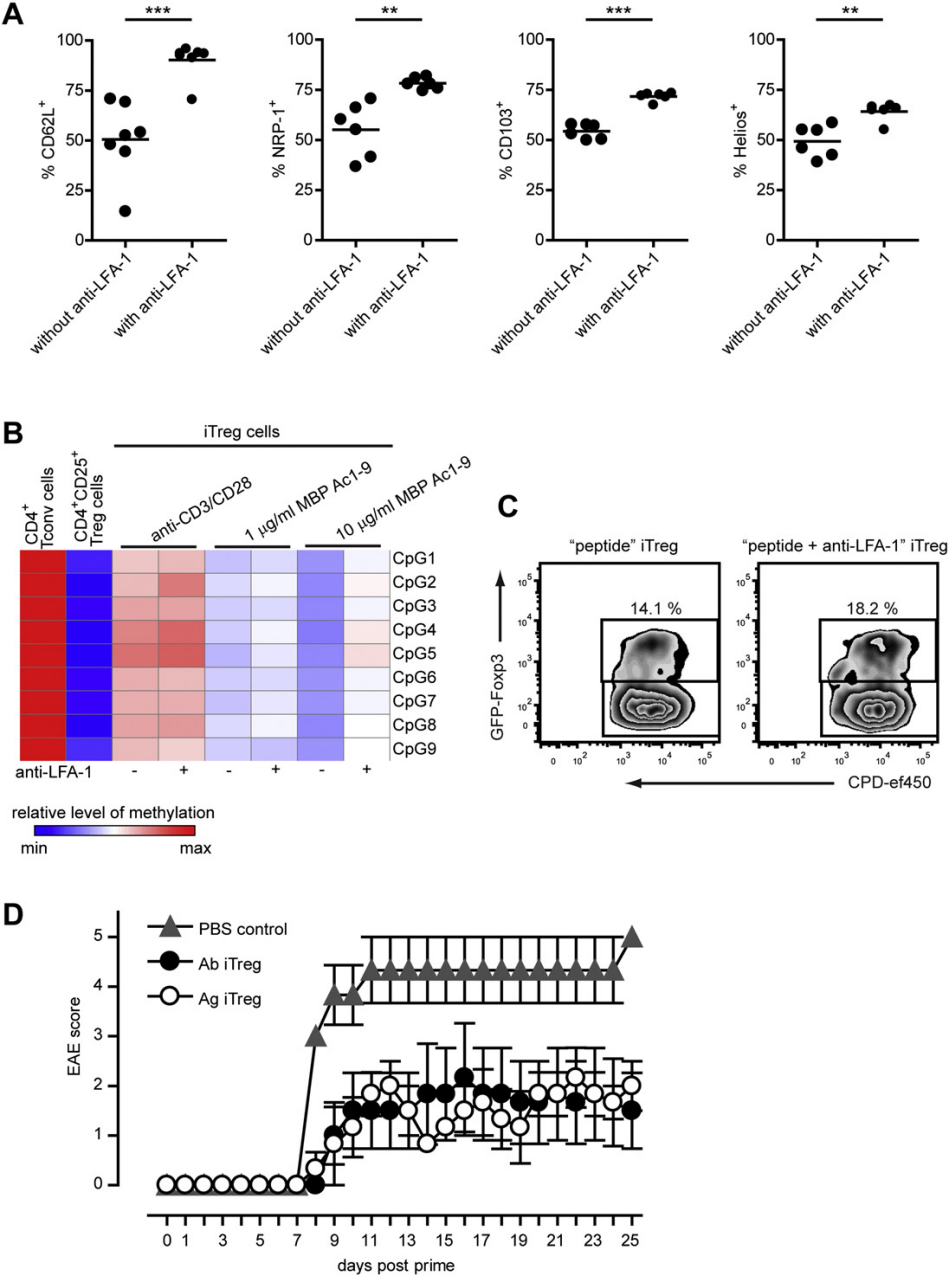
Anti-LFA-1 treatment alters the phenotype but not the stability or function of iTreg cells. A: Expression of CD62L, Neuropilin-1 (NRP-1), CD103 and Helios on Foxp3^+^ iTreg cells after 7-day differentiation culture with 1 μg/ml MBP Ac1-9 in the presence or absence of 10 μg/ml anti-LFA-1. Pooled data, n = 6–7 per condition. **P < 0.01, *** P < 0.001,unpaired, 2-tailed t-test. B: Analysis of the relative level of methylation of 9 CpG in the foxp3 CNS2 region of naive CD4^+^ T cells, CD4^+^CD25^+^ splenic Treg cells or iTreg cells generated under various conditions, in the presence or absence of 10 μg/ml soluble anti-LFA-1 (all Tg4). Blocks in each column represent the mean values of three replicate samples. C: GFP-Foxp3 retention in Tg4 Foxp3^gfp^ iTreg cells in vivo. FACS-sorted iTreg cells labeled with CPD-ef450 were transferred to Tg4 recipients. Mice were challenged with a high affinity variant of MBP Ac1-9 48 h post transfer. 72 h post challenge iTreg cells retrieved from the spleen were analyzed for proliferation and Foxp3 retention by FACS. One experiment representative of 3 is shown. D: Tg4 mice received 5 × 10^6^ Tg4 iTreg cells generated using either stimulation with 1 μl/ml MBP Ac1-9 and soluble anti-LFA-1 (Ag iTreg) or anti-CD3 and anti-CD28 (Ab iTreg) in PBS, or PBS only, i.p. 3 days prior to EAE induction with MBP Ac1-9 in CFA and were monitored daily for disease. n = 3 per group, representative of 2 similar experiments.

As shown above, anti-LFA-1 treatment enhances the efficacy of antigen-mediated iTreg cell differentiation but the question remained whether this technique resulted in iTreg cells not only of higher purity but also of equal or greater functionality. First, the effect of anti-LFA-1 on the iTreg cell phenotype was assessed. Fig. 3A shows that CD62L, Neuropilin-1 (NRP-1), CD103 and Helios, molecules commonly associated with Treg cell function, were all expressed on a greater proportion of iTreg cells differentiated in the presence of anti-LFA-1 than in its absence. Next, the effect of LFA-1 blockade on the stability of Foxp3 expression was assessed since instability may be associated with undesirable immune responses mediated by iTreg cells that have reverted to an effector function. The stability of Foxp3 expression is regulated primarily by demethylation of the CNS2 region of the foxp3 promoter (Zheng et al., 2010). In our model, iTreg cells generated either with peptide and APCs or with plate-bound anti-CD3 and anti-CD28 demonstrated a level of methylation intermediate between that of Tconv cells and CD4^+^CD25^+^Foxp3^+^ splenic Treg cells (Fig. 3B). The addition of soluble anti-LFA-1 during differentiation did not lower the level of methylation and in the presence of the higher 10 μg dose of MBP Ac1-9 may have even impaired demethylation. To determine whether iTreg cells stably expressed Foxp3, iTreg cells were generated, in the presence or absence of anti-LFA-1, from naive T cells of male Tg4 Foxp3^gfp^ mice (Verhagen et al., 2013a), FACS-sorted to high purity and, after labeling with Cell Proliferation Dye-ef450, transferred intraperitoneally to sex-matched recipient Tg4 mice. After 48 h, these mice were challenged with a single dose of a high MHC II affinity variant of the MBP Ac1-9 peptide (Ac-ASQYRPSQR). 72 h post-challenge the transferred iTreg cells were recovered from the spleen and analyzed for retention of Foxp3 expression, which had diminished greatly, regardless of the addition of anti-LFA-1 during the iTreg cell differentiation culture (Fig. 3C). Although the instability in this model may be augmented by the GFP-Foxp3 fusion protein (Verhagen et al., 2013a), the in vivo stability data are in line with the CNS methylation analysis and indicate that LFA-1 blockade during differentiation does not offer iTreg cells greater stability of Foxp3 expression.

Despite a lack of CNS2 demethylation at levels akin to naturally occurring CD4^+^CD25^+^ Treg cells and stability of Foxp3 expression, adoptive transfer of antigen-specific iTreg cells delayed the progression of CNS autoimmune disease. To demonstrate this, Tg4 mice received 5 × 10^6^ iTreg cells intraperitoneally 3 days prior to EAE induction with MBP Ac1-9 in CFA. As shown in Fig. 3D, Tg4 iTreg cells generated using antigenic stimulation and anti-LFA-1 provided equal levels of protection compared to anti-CD3 + anti-CD28-induced Tg4 iTreg cells of similar purity, i.e. > 90%.

Overall, we demonstrate here that functional iTreg cells can be differentiated from self-antigen-specific Tconv cells by in vitro stimulation with peptide in the presence of IL-2 and TGF-β. Importantly, the efficacy of induction of Foxp3 expression is enhanced by the blockade of LFA-1 with monoclonal antibody. This will facilitate the differentiation of greater numbers of antigen-specific iTreg cells at high purity, thereby improving the feasibility, efficacy and safety of iTreg cell-based immunotherapy.
